# Electrical and Recombination Properties of Polar Orthorhombic κ-Ga_2_O_3_ Films Prepared by Halide Vapor Phase Epitaxy

**DOI:** 10.3390/nano13071214

**Published:** 2023-03-29

**Authors:** Eugene B. Yakimov, Alexander Y. Polyakov, Vladimir I. Nikolaev, Alexei I. Pechnikov, Mikhail P. Scheglov, Eugene E. Yakimov, Stephen J. Pearton

**Affiliations:** 1 Institute of Microelectronics Technology and High Purity Materials, Russian Academy of Sciences, 6 Academician Ossipyan Str., Chernogolovka 142432, Russia; 2 Department of Semiconductor Electronics and Physics of Semiconductors, National University of Science and Technology MISiS, 4 Leninsky Avenue, Moscow 119049, Russia; 3 Perfect Crystals LLC, 28 Politekhnicheskaya Str., St. Petersburg 194064, Russia; 4 Ioffe Institute, 26 Polytekhnicheskaya Str., St. Petersburg 194021, Russia; 5 Department of Materials Science and Engineering, University of Florida, Gainesville, FL 32611, USA

**Keywords:** κ-Ga_2_O_3_, rotational nanodomains, electrical properties, deep traps, DLTS, EBIC, cathodoluminescence

## Abstract

In this study, the structural and electrical properties of orthorhombic κ-Ga_2_O_3_ films prepared using Halide Vapor Phase Epitaxy (HVPE) on AlN/Si and GaN/sapphire templates were studied. For κ-Ga_2_O_3_/AlN/Si structures, the formation of two-dimensional hole layers in the Ga_2_O_3_ was studied and, based on theoretical calculations, was explained by the impact of the difference in the spontaneous polarizations of κ-Ga_2_O_3_ and AlN. Structural studies indicated that in the thickest κ-Ga_2_O_3_/GaN/sapphire layer used, the formation of rotational nanodomains was suppressed. For thick (23 μm and 86 μm) κ-Ga_2_O_3_ films grown on GaN/sapphire, the good rectifying characteristics of Ni Schottky diodes were observed. In addition, deep trap spectra and electron beam-induced current measurements were performed for the first time in this polytype. These experiments show that the uppermost 2 µm layer of the grown films contains a high density of rather deep electron traps near E_c_ − 0.3 eV and E_c_ − 0.7 eV, whose presence results in the relatively high series resistance of the structures. The diffusion length of the excess charge carriers was measured for the first time in κ-Ga_2_O_3_. The film with the greatest thickness of 86 μm was irradiated with protons and the carrier removal rate was about 10 cm^−1^, which is considerably lower than that for β-Ga_2_O_3_.

## 1. Introduction

Ga_2_O_3_ is a wide-bandgap semiconductor that is currently creating great interest due to its extremely attractive properties, which can be utilized in power devices and solar-blind far-UV photodetectors [1,2,3]. Like many other wide-bandgap materials, Ga_2_O_3_ is characterized by the existence of a great number of polymorphs, among which the most important are the thermodynamically stable monoclinic β-Ga_2_O_3_ with a bandgap of 4.8 eV, metastable polytypes of rhombohedral α-Ga_2_O_3_ with bandgaps of 5.2–5.3 eV, and orthorhombic κ-Ga_2_O_3_ with a bandgap close to 4.6 eV. To a lesser extent, γ-Ga_2_O_3_, with the structure of a defect spinel [1,2,3], is also currently being studied. This is believed to be formed during a transitory phase when α-Ga_2_O_3_ or κ-Ga2O_3_ [4] is heated up, or when the heavy ion implantation of β-Ga_2_O_3_ is carried out [5]. Researchers are mainly interested in the stable β-Ga_2_O_3_ polymorph. This material can be grown from the melt in the form of high-quality crystalline bulk crystals, a process that advantageously distinguishes it from the more established GaN or SiC material systems [1,2]. Various versions of epitaxial growth on native β-Ga_2_O_3_ substrates have been successfully developed, which is an advancement that allows for the growth of epitaxial films and heterostructures that are of very good quality and are suitable for the fabrication of power devices and photodetectors with promising performance. The state of growth of bulk and epitaxial β-Ga_2_O_3_ films and structures, the fabrication of various devices, and experimental and theoretical studies on the defects and impurities that exist in β-Ga_2_O_3_ have been described in several recent books and reviews [2,6,7,8,9]. To summarize this progress, β-Ga_2_O_3_ devices are currently characterized by very high operating voltages that are rapidly approaching the theoretically predicted limits based on the estimated electric breakdown field of 8 MV/cm. They also exhibit good switching speeds due to the high saturation velocity of electrons. Photodetectors show excellent photosensitivity in the far-UV-C spectral range, with a high rejection ratio for photosensitivity in the visible and near-UV range; this is owing to the very large bandgap and the reasonably low density of defects, which create centers with deep levels in the gap [1,2,3,4,5,6,7,8,9,10]. Both n-type bulk crystals and epi-layers can be controllably grown with doping over a wide range of concentrations, as well as via semi-insulating, by incorporating Fe, Mg or N acceptors [2]. Bulk crystals and epi-structures of β-Ga_2_O_3_ are commercially available (see e.g., [11]). Prototype devices of various types with characteristics that exceed those based on more mature wide-bandgap materials have been demonstrated by many research groups and are approaching the stage at which they will become commercially available [1,2,3,4,5,6,7,8,9,10]. The remaining problems in this materials system are related to the absence of defects and impurities that are suitable for viable p-type doping, the low thermal conductivity of β-Ga_2_O_3_, considerable anisotropy related to low levels of lattice symmetry, and the relatively low electron mobility [1,2,3,4,5,6,7,8,9,10].

The α-Ga_2_O_3_ polymorph is isomorphous with α-Al_2_O_3_ (sapphire). This makes it possible to grow the material on inexpensive, large-diameter, and high-quality sapphire substrates. The bandgap of α-Ga_2_O_3_ is higher than β-Ga_2_O_3_, which potentially allows even higher breakdown fields. The lattice symmetry of the former is higher and there exists a host of p-type corundum structure metal oxides that are compatible with the growth of heterojunctions and solid solutions with α-Ga_2_O_3_ [12]. The preferred growth techniques for the deposition of α-Ga_2_O_3_ films are Halide Vapor Phase Epitaxy (HVPE) and mist CVD [12,13,14], with research also being conducted using Molecular Beam Epitaxy (MBE) [15]. The drawbacks of the corundum polymorph system are relatively large lattice mismatch of α-Ga_2_O_3_ with sapphire, giving rise to a dislocation density over 10^9^ cm^−2^, and the relatively low thermal stability of α-Ga_2_O_3_ (stable up to temperatures only slightly above 600 °C). In addition, there is competition with the other metastable polymorph, κ-Ga_2_O_3_, during growth on sapphire [14]. The high dislocation density problem can be alleviated using strain-relieving α-(Al_x_Ga_1-x_)_2_O_3_ buffers in mist CVD [16], or via growth using magnetron-sputtered α-Cr_2_O_3_ buffers in HVPE [17,18]. A radical decrease in the dislocation density down to 10^6^–10^7^ cm^−2^ can be achieved via the application of the Epitaxial Lateral Overgrowth (ELOG) technique [19]. The problem of mixed polytypes can be mitigated by carefully choosing the deposition temperatures and by applying all the strain-relief dislocation density methods. These problems are less severe when growth is carried out on sapphire planes other than the basal plane. The thermal stability that exists in MBE growth has been shown to greatly increase (up to about 900 °C) when masking the surface with α-(Al_x_Ga_1−x_)_2_O_3_ capping layers [20]. The defect studies in α-Ga_2_O_3_ are not as advanced as in β-Ga_2_O_3_, but some work on the theory of defects has been started [21]. In addition, some experimental studies on defect structures and the properties of defects have been conducted and summarized in recent reviews [9,22]. α-Ga_2_O_3_-based power rectifiers and solar-blind photodetectors with promising properties have been demonstrated [23,24].

The main interest in the κ-Ga_2_O_3_ polymorph is stimulated by the fact that it alone among the Ga_2_O_3_ polymorphs displays a high degree of electron spontaneous polarization and prominent ferroelectric properties [25,26]. The spontaneous polarization of κ-Ga_2_O_3_ is several times higher than that of AlN, so it may be possible to obtain very high densities of Two-Dimensional Electron Gas (2DEG) or Two-Dimensional Hole Gas (2DHG) in heterojunctions involving κ-Ga_2_O_3_. These heterojunctions could be used as channel layers in Field Effect Transistors (FETs) [27]. The formation of 2DEG and 2DHG at interfaces of heterojunctions is created by combining κ-Ga_2_O_3_ with different polar and nonpolar materials, such as polar basal plane GaN and AlN, or nonpolar m-plane AlN, which have been theoretically predicted in several papers [28,29]. In our earlier work [30], we reported the experimental observation of a 2DHG at the heterointerface of κ-Ga_2_O_3_ films with basal plane (0001) AlN on Si(111). It has also been suggested that the considerable piezo-electric polarization of κ-Ga_2_O_3_ could be employed in RF resonators and modulators [31].

Unfortunately, the crystalline quality of the κ-Ga_2_O_3_ films grown using various techniques is plagued by the tendency of the material to grow in the form of small (5–10 nm) nanocrystals that are rotated by 120° around the growth axis [4,32,33]. The presence of these rotational domains initially leads to them being incorrectly assigned to the hexagonal symmetry and group symmetry of P63mc, and regarding the material as ε-Ga_2_O_3_ [25] instead of orthorhombic Pna21 κ-Ga_2_O_3_, which is correct. Thus, although a growth of single-domain films has been reported [34], in most cases, κ-Ga_2_O_3_ can be considered to be a nanocrystalline material. The boundaries between these rotational domains present an obstacle to the current flow in the growth plane and, for a long time, have prevented the research of the electrical properties of κ-Ga_2_O_3_ films; however, more recently, heterostructures and quantum wells of κ-(Al_x_Ga_1−x_)_2_O_3_/κ-Ga_2_O_3_ with different Al mole fractions and hence different band structures have been demonstrated, and vertical NiO/κ-Ga_2_O_3_ structures have been successfully fabricated by pulsed laser deposition [35,36]. This major disadvantage impairing the practical use of κ-Ga_2_O_3_-based structures and devices was partly overcome in [19] by employing ELOG growth on sapphire substrates masked with thin TiO_2_ and patterned with either stripes or dots of SiO_2_; this facilitated the formation of single-domain κ-Ga_2_O_3_ films with good crystalline quality using HVPE. In addition, the authors of [32,34] were able to prepare single-domain κ-Ga_2_O_3_ films using mist CVD growth on the ε-GaFeO_3_ substrate. However, neither work detailed the electrical characterization of the κ-Ga_2_O_3_ films grown.

The formation of κ-Ga_2_O_3_ in HVPE growth can be achieved when using sapphire, GaN, AlN and SiC substrates [30,37]. With growth on sapphire, the formation of κ-Ga_2_O_3_ is stimulated by deposition on the patterned substrates for which κ(ε)-Ga_2_O_3_ is preferentially formed in the valleys of the patterned sapphire; meanwhile, α-Ga_2_O_3_ grows on the tops of the hillocks in the pattern [14]. By choosing deposition temperatures above ~600 °C, it is possible, in principle, to obtain continuous κ-Ga_2_O_3_ films [14]. The electrical properties, deep trap spectra and luminescence spectra of composite α-Ga_2_O_3_/κ-Ga_2_O_3_ films with α-Ga_2_O_3_ on top and grown by HVPE on patterned sapphire at temperatures below 600 °C have been studied [14]. It has been noted that the effective conductivity of α-Ga_2_O_3_ in such structures is, in general, higher than that for films deposited on planar sapphire; however, the reason for this is not currently understood.

In experiments with HVPE growth on other substrates, it has been pointed out that the formation of κ-Ga_2_O_3_ is favored by a high degree of elastic strain [38]. Thus, growth on heavily lattice mismatched hexagonal GaN, AlN and SiC has resulted in the formation of films of pure κ-Ga_2_O_3_. It has been also suggested that high levels of strain in heavily disordered β-Ga_2_O_3_ when exposed to high-dose ion implantation could give rise to the formation of defective κ-Ga_2_O_3_ layers [38,39]; however, this hypothesis has been challenged in recent papers [5,40].

With the HVPE growth of κ-Ga_2_O_3_, it could be expected that, by increasing the thickness of the grown layers, one could achieve the state in which the process switches to a step-flow preferentially lateral growth mode that favors an improvement in the overall crystalline quality and facilitates a single-domain structure similar to that reported in ELOG growth. It is well known that, in the GaN system, this approach serves as the basis of high-quality free-standing substrates and bulk crystals. Until recently, such thick κ-Ga_2_O_3_ films have not been successfully grown, but lately we have achieved success on that front [41,42]. This has allowed, for the first time, the detailed characterization of the electrical, luminescent and recombination properties of such Ga_2_O_3_ films and their deep trap spectra with an improvement in the crystalline quality. In this paper, we briefly describe the growth methods, structural characterization, electrical characterization, microcathodoluminescence (MCL) spectra, deep trap spectra, photosensitivity, and diffusion length measurements for such κ-Ga_2_O_3_ films.

The main objective of this article was to collect together the results of electrical and deep traps studies on κ-Ga_2_O_3_ grown under different conditions and the possible role of Halide Vapor Phase Epitaxy (HVPE) growth on alien substrates that favor the formation of κ-Ga_2_O_3_. Although the properties of κ-Ga_2_O_3_ that are favorable to the fabrication of efficient high-power devices were recognized some time ago, actual studies of the effects of HVPE growth conditions on achieving films with reasonably good crystalline quality and in-plane electrical conductivity have been scarce; in addition, studies on these films’ deep traps spectra, photosensitivity, recombination properties, the effects of credible demonstrations of 2D conductivity when predicted by theoretical modeling, and the effects of irradiation compared to other Ga_2_O_3_ polymorphs, have been very scarce. In this paper, we have gathered together the results that have been recently achieved on these fronts in our research group. Although multiple problems still remain to be solved, it has clearly become evident that the HVPE growth of thick κ-Ga_2_O_3_ films with electrical properties that are suitable for use in practical applications is approaching the state at which it will become possible to grow device-quality films; in the meantime, the effects of 2D conductivity can indeed be observed, although methods have to be devised in order to capitalize on these effects in real-life devices.

## 2. Materials and Methods

### 2.1. Growth

The κ-Ga_2_O_3_ films described in this work were grown using HVPE in a hot wall atmospheric pressure reactor, allowing growth on substrates up to 2 inches in diameter at temperatures up to 1200 °C [43]. GaCl vapor that was synthesized in situ in the reactor via the reaction of high-purity metallic Ga with gaseous HCl served as the Ga precursor, while spectrally clean O_2_ served as the oxygen precursor. The carrier gas was Ar, and Sn was used as the n-type dopant. The mole flow ratio of the VI/III components was 3 and the growth temperature was 570 °C, at a growth rate of 3 μm/h. The experiments discussed in this paper were performed on AlN(0001) (0.5 μm)/Si(111) templates that were prepared using HVPE and on GaN(0001) (4 μm)/sapphire templates that were prepared using HVPE in a separate reactor. The thickness of the κ-Ga_2_O_3_ film grown on AlN/Si was 2.1 μm, and the thickness of the κ-Ga_2_O_3_ films grown on GaN/sapphire templates varied from 2 μm to 86 μm. For the thicker films, growth was performed in several consecutive runs, which alleviated strain and prevented the film from separating from the substrate.

The morphology of the grown films was analyzed by observation in the secondary electron mode (SE) of the Scanning Electron Microscope (SEM) JSM-6490 (JEOL, Tokyo, Japan). The structural characterization of the Ga_2_O_3_ layers was performed using X-ray diffraction in the θ-2θ mode and double crystal measurements of the Full Width at Half Maximum (FWHM) of the symmetric and asymmetric Ga_2_O_3_ X-ray reflections, by taking measurements of the film bowing radius using low-angle X-ray diffraction [42,43].

### 2.2. Characterization of Electrical Properties, Deep Trap Spectra, Photocurrent and Photocapacitance Spectra, and Recombination Properties

Ohmic contacts of the samples were obtained via the e-beam evaporation of Ti/Au (20 nm/80 nm). Schottky contacts approximately 1 mm in diameter were deposited via the e-beam evaporation of Ni (20 nm) through a shadow mask. These contacts were semi-transparent to visible and UV light. No contact annealing was performed, which introduced no serious problems for current and capacitance measurements.

The samples were characterized by taking current-voltage (I-V), capacitance-voltage (C-V), capacitance-versus frequency (C-f), and admittance spectra measurements in the temperature range of 77 to 500 K. These experiments were performed in the dark and also under illumination using a set of high-power light-emitting diodes (LEDs) with wavelengths in the range of 365 to 940 nm; this was complemented by taking measurements using deep UV LEDs with wavelengths of 277 nm and 259 nm, with an optical power that was a hundred times lower. Using the former LEDs meant that the optical power density could be varied from a few mW/cm^2^ to over 250 mW/cm^2^; the optical output power density of the 277 UV LED was lower, up 15 mW/cm^2^, which was up to 1.2 mW/cm^2^ for the 259 nm LED. The 365–940 nm wavelength LEDs provided below-bandgap excitation, and the 277 nm and 259 nm LEDs produced the approximately band edge and above bandgap optical excitation of our k-Ga_2_O_3_ films, with a bandgap of 4.6 eV. In addition, measurements were also taken after illumination to determine the existence of centers with a high barrier for the capture of electrons [44,45]. The frequency range in which meaningful C-V profiling in the dark and under illumination could be performed was determined using the C-f measurements. If these showed several steps in frequency, taking C-V measurements at each step meant that the spatial profiles of the centers responding to the specified probing frequency could be calculated, thus contributing to the capacitance. This meant that the profiles of the centers responding to the frequency corresponding to the chosen step could be obtained. Then, taking measurements of the temperature dependence of capacitance at various frequencies and of AC conductance *G* (or the capacitance derivative on temperature d*C*/d*T*) meant that the depth of the centers and the capture cross-section of electrons on them could be determined by examining the shift in the temperature of the steps in the capacitance, or by examining the shift in the peaks in the conductance with frequency (this approach is called Admittance Spectroscopy (AS) [46]). In building such spectra, the AC conductance is commonly normalized by the angular frequency ω = 2π*f* (*f* is the measured frequency) [46].

Deep trap spectra were obtained from DLTS measurements with electrical (DLTS) or optical (ODLTS) excitation [46,47], with the latter using the same set of LEDs as described above. All experiments were performed using a custom-built setup [44], which involved an E4980A LCR meter (KeySight Technologies, Santa Rosa, CA, USA, frequency range of 20 Hz–1 MHz), a B2902A voltage/current source/meter (KeySight Technologies, USA), a Cryotrade (Russia) cold-finger liquid nitrogen cryostat, which allows the measurement temperature to be maintained in the 77 to 500 K temperature range with an accuracy greater than 0.1 K and the temperature in the measurements to be swept with a controlled temperature sweep range (commonly, sweep rates close to 1–2 K/minute were used). The E4980A LCR meter was used for C-V profiling and admittance spectra measurements; this was used together with the external pulse generator 33500B (KeySight Technologies, USA) to take DLTS measurements. For ODLTS measurements, the optical pulsing was provided by the B2902A current–voltage source/meter (KeySight Technologies, USA), which drove the LED used for excitation. The same device was also used for I-V measurements and for C-V, I-V, C-f, admittance measurements with illumination. In the course of taking the DLTS/ODLTS measurements, the capacitance transient was monitored and digitized. The relaxation curve measured at each temperature step was digitized and processed either by the usual two-gate approach (the mode was used for monitoring the results during the measurements) [47], by Laplace DLTS analysis [47], or by analyzing the actual waveform of the capacitance transient. The advantage of this system is that measurements can be taken at any frequency in the 1 kHz–1 MHz range, which means that capacitance freeze-out and high series resistance effects can be accounted for. The sensitivity of the system was 5 × 10^−4^ of the density of shallow donors [48]. The drawback of the system is that the digitization of the capacitance relaxation curves can be performed only with relatively long time steps of 15 ms for a probing frequency of 1 MHz and of 40 ms for 1 kHz. This limits the highest emission rate that can be set in our DLTS experiments to 46.1 s^−1^ at 1 MHz, which is lower than that for advanced commercial DLTS machines based on fast capacitance bridges. The use of this system, instead of commercial DLTS systems with a fixed probing frequency of 1 MHz, proved to be largely instrumental in enabling deep traps spectra analysis to be performed in the k-Ga_2_O_3_ films described in the next section; this is because the series resistance of these films, even after all improvements, remained quite high.

The bandgap of κ-Ga_2_O_3_ is large, so DLTS/ODLTS measurements allowed only the parameters of the deep electron and hole traps in the energy bands that adjust the conductance and valence bands to be probed. This corresponds to the states that lie between the edge of the conductance and valence bands, and ~*E_c_* − (1.2–1.5) eV or *E_v_* + (1.2–1.5) eV, because of the limitations on the temperatures to which the spectra measurements can be extended. This, in turn, limits the lengths of the capacitance/current relaxation times that can be probed at high measurement temperatures [45]. For very wide bandgap semiconductors, such as Ga_2_O_3_, an alternative approach consists of combining photocapacitance (PC) spectra measurements and C-V profiling with monochromatic light illumination (LCV measurements) [49] in order to characterize deep traps that are not accessible to DLTS/ODLTS probing. In our experiments, such measurements were performed by using the high-power LEDs to build the PC and LCV spectra. The deep traps that had a high barrier for the capture of electrons could be pinpointed by measuring the PC and C-V characteristics after the illumination was switched off and after checking whether the persistent photocapacitance after the termination of light could be quenched by the application of a high forward current. The persistence of the capacitance after illumination was due to the absence of free electrons in the Space Charge Region (SCR) of the Schottky diode in the dark, so the excessive charge on the traps could be relieved only by the thermal emission of non-equilibrium carriers from the trap or via recombination with the charge carriers provided by the dark current of the Schottky diode. Both processes have long time constants. However, if one applies a large forward current, it will remove the excess charge on the traps quite rapidly, unless the traps possess a high barrier for the capture of electrons [45]. This approach allows the traps with and without barriers for capture to be discriminated [45,49]. In principle, performing such measurements at different temperatures and with different injection pulse lengths allows both the barrier capture height and the capture cross-section of majority carriers for minority carrier traps to be determined, as has been demonstrated for n-GaN [45]; however, for Ga_2_O_3_, such experiments have yet to be conducted.

Microcathodoluminescence (MCL) spectra measurements were performed in a JSM-6490 (Jeol, Japan) SEM at room temperature using the MonoCL3 (Gatan, Abingdon, UK) system with a Hamamatsu photomultiplier as a detector. In most experiments, the CL measurements were carried out with a beam energy of *E_b_* of 10 keV and a beam current of *I_b_* of about 1 nA. The experimentally measured broad MCL spectra were deconvoluted into a set of Gaussian bands fitting the experiment and allowing the individual radiative recombination bands and their relative intensities to be determined.

The recombination rate of the excess carriers was characterized by Electron Beam Induced Current (EBIC) measurements. The experimental setup and the treatment procedure were described in more detail previously [50,51]. The EBIC studies were carried out in the JSM-840 SEM (Jeol, Japan) scanning electron microscope at room temperature with a beam energy *E_b_* in the range of 3 keV to 38 keV and a beam current of 10^−10^. A Keithley 428 current amplifier was used in the EBIC measurements. Briefly, the procedure of estimating the diffusion length consisted of fitting the experimentally measured and collected current *I_c_* dependence on beam energy *E_b_* to the calculated one. ρ

(1) $$I_{c}=e\int_{t_{m}}^{\infty }h(z)\psi (z,L)dz,$$

where *e* is the electron charge; *t_m_* is the metal thickness; *h*(*z*) is the depth-dependent excess carrier generation rate calculated using the Monte Carlo algorithm; *z* is the depth; *L* is the diffusion length; and ψ(*z*, *L*) is the collection probability, which can be calculated using the solution of a homogeneous diffusion equation [52]. For the *h*(*z*) calculation, the expression obtained for β-Ga_2_O_3_ in [51,53] was used:

(2) $$h(z)=\frac{1.603}{R}exp[-A{\left(\frac{z}{R}-0.22\right)}^{2}],$$

where *R* (nm) = 7.34·*E_b_* (keV)^1.75^ and $A=\left\{ \begin{array}{l} 12.86,z<0.22\cdot R\\ 3.97,z\geq 0.22\cdot R \end{array}\right.$.

## 3. Results and Discussion

### 3.1. Thin κ-Ga_2_O_3_ Films on AlN/Si Templates: Electrical Properties and Deep Traps

**Figure 1:**
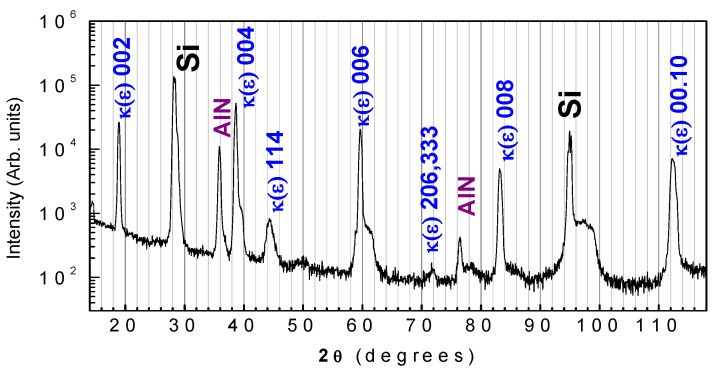
θ-2θ scan of the Ga_2_O_3_/AlN(0001)/Si(111) sample.

**Figure 2:**
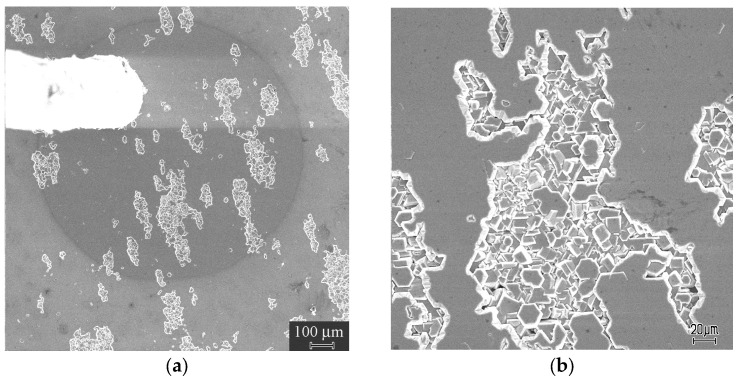
Secondary electron image of thin κ-Ga_2_O_3_ film with the Schottky diode for electrical measurements (**a**) and a fragment of the image with the higher magnification (**b**).

The κ-Ga_2_O_3_ film in question was grown on a AlN(0001) (unintentionally doped)/Si(111) (high-resistivity *p*-type) template prepared using HVPE in another HVPE reactor dedicated to the growth of III-Nitrides. The thickness of the AlN film was 0.6 μm and the resistivity of the Si substrate was 5000 Ωcm. The κ-Ga_2_O_3_ film was grown at 530 °C and the overall thickness of the film was 2.1 μm. The top ~0.6 μm of the film was lightly doped with Sn, while the bottom part adjacent to AlN was nominally undoped. The θ-2θ X-ray pattern taken from the Ga_2_O_3_ side clearly shows that the grown Ga oxide film is the orthorhombic κ-Ga_2_O_3_ polymorph with a growth surface in the (001) orientation (Figure 1). The FWHM of the High-Resolution X-ray Diffraction (XRD) rocking curve for the (002) symmetric reflection of κ-Ga_2_O_3_ was actually quite high, being 1.1 degrees; meanwhile, the FWHM of the (0002) AlN reflection was 1.8 degrees, and the FWHM of the Si(111) reflection was 72 arcseconds. Thus, the crystalline quality of the κ-Ga_2_O_3_ film and of the AlN film was low because of the strong lattice mismatch (compare this HRXRD for κ-Ga_2_O with the data for thick κ-Ga_2_O_3_ films on GaN in the next section). In SEM images, regions with an accumulation of microcrystals with hexagonal faceting are seen; these correlate well with the presence of rotational nanodomains (Figure 2). As shown below, the number of such regions essentially decreases with the film thickness.

**Figure 3:**
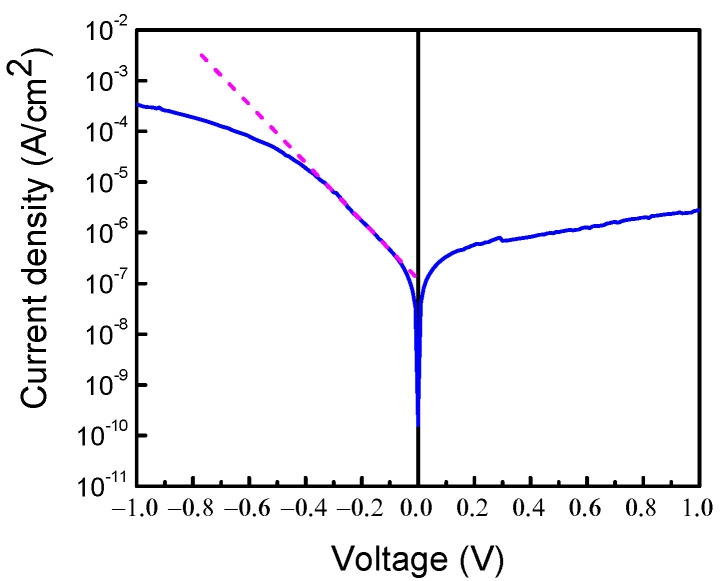
Room temperature I-V characteristic of the sample.

**Figure 4:**
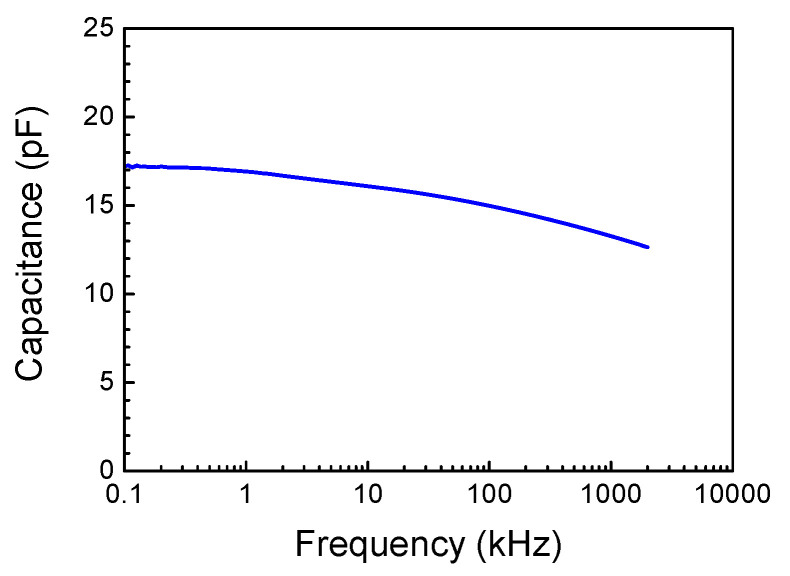
Room temperature C-f characteristic of the sample.

**Figure 5:**
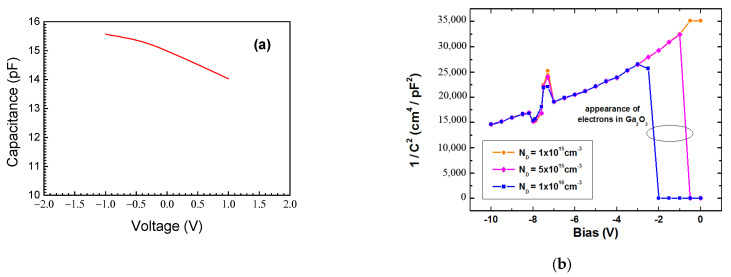
(**a**) Experimental C-V characteristic, (**b**) modeled 1/C^2^ versus V for three donor concentrations.

The electrical properties of the Schottky diode prepared on the structure are illustrated in Figure 3, showing the room temperature I-V characteristic. Figure 4 also displays the room temperature C-f characteristic and Figure 5a shows the C-V characteristic measured at 100 kHz. In these Figures, the sign of the applied voltage corresponds to that applied to the Schottky diode metal. It can be immediately seen that the electrical properties of the Schottky diode prepared on the κ-Ga_2_O_3_ film are unusual in that the current and capacitance at the negative voltage on the Schottky diode are much higher than those for the positive bias. This is opposite to what one expects from the Schottky diode prepared on n-type material; however, this would be expected for the Schottky diode on a p-type film. This is not a consequence of the Ohmic contacts misbehaving, since the I-V characteristic measured between the two Ohmic contacts was perfectly linear. Additionally, it is well known that in Ga_2_O_3_, there are no dopants suitable for obtaining p-type conductivity because the valence band of Ga_2_O_3_ is formed predominantly from the 2*p* states of the oxygen atom and that it lies very deep in respect to the level of vacuum [54].

**Figure 6:**
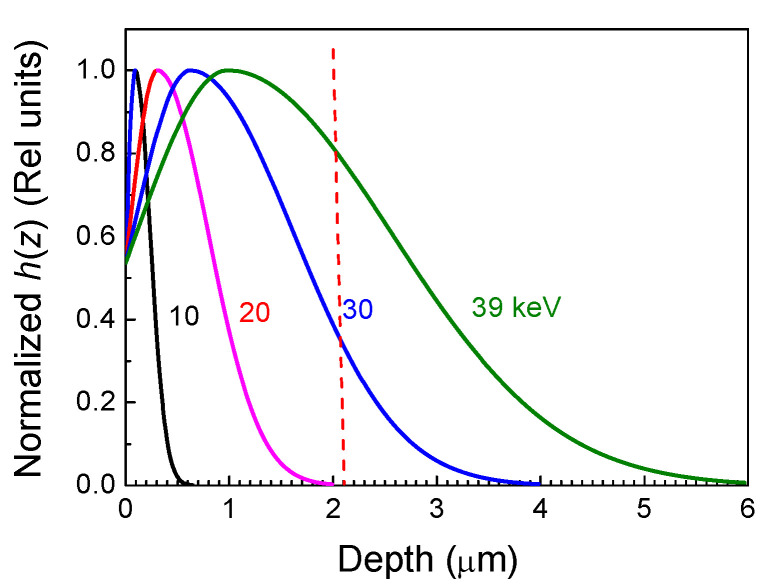
Normalized carrier generation rate calculated for different probing beam energies. The dashed red line shows the film thickness.

Another unusual feature was observed in EBIC measurements on these Schottky diodes. Namely, the prominent EBIC signal for the structure appeared only when the energy of the probing electron beam in SEM became higher than 30 keV, corresponding to the peak in the excessive carrier generation rate dependence on *z*, *h*(*z*); this reached the interface between the κ-Ga_2_O_3_ and the AlN substrate. Figure 6, illustrating this point, shows the *h*(*z*) dependences for four beam energies calculated using Equation (2).

**Figure 7:**
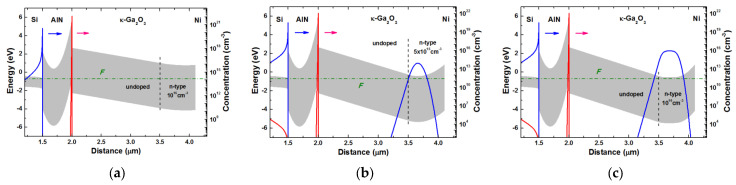
Calculated band diagrams and profiles of holes (red lines) and electrons (blue lines) in the κ-Ga_2_O_3_/AlN(0001)/Si(111) heterojunctions with three different donor concentrations (**a**) 1 × 10^15^ cm^−3^, (**b**) 5 × 10^15^ cm^−3^, (**c**) 1 × 10^16^ cm^−3^; the arrows show the spontaneous polarization vector direction. Electron distribution is shown by red lines and that of holes by blue ones.

This observation suggests that the excess carriers are mostly collected by the second junction located near the AlN/κ-Ga_2_O_3_ interface due to a rectifying contact between the interfacial p-layer in Ga_2_O_3_ and the n-type, or highly resistive n-Ga_2_O_3_. The most likely reason for this is revealed by theoretical modeling, which takes into account the high spontaneous polarization in (0001)AlN and (001) κ-Ga_2_O_3_. Such modeling, using the package FETIS [55] adapted to κ-Ga_2_O_3_, was performed in Ref. [30] by Dr. S. Yu. Karpov of STR-Soft-Impact, Ltd., St. Petersburg. The parameters of the materials used in the calculation can be found in the Supplementary Material to [30]. Figure 7 illustrates the results of this modeling, showing that, indeed, the high-density 2DHG interfacial layer is formed at the κ-Ga_2_O_3_/AlN heterojunction. In addition, the p-Ga_2_O_3_/n-Ga_2_O_3_ junction is switched in series with the top Schottky diode with n-Ga_2_O_3_. As calculations show, the ability to detect this second heterojunction is critically dependent on the shallow donor concentration in the k-Ga_2_O_3_ film near the surface. When the concentration is 10^15^ cm^−3^, the film at zero bias is depleted of electrons and the structure is a leaky metal–insulator–semiconductor device. For higher donor doping, the top Schottky diode becomes operable and the results of modeling predict the collapse of detectable p-type behavior, as shown in Figure 5b. The model also requires the existence of a way to transport charge carriers via the bulk of the depleted κ-Ga_2_O_3_ film between the Ohmic contact and the interfacial p-n junction. This path could be provided by hopping via the high dislocation density in the heavily lattice mismatched κ-Ga_2_O_3_/AlN/Si structure, as revealed by X-ray measurements, or via the boundaries of rotational nanodomens.

The structure demonstrates the possibility of forming a high-density 2DHG at the interface between κ-Ga_2_O_3_ and AlN. Such a structure, however, is of limited practical use because of the narrow doping region in which the effect can be observed. The high resistivity of AlN means that an unorthodox current flow path must be relied upon via extended states and that there is difficulty in forming a good Ohmic contact to AlN in the mesa structure. However, modeling suggests that similar “polarization” p-type doping should be observed in κ-Ga_2_O_3_/GaN heterojunctions, which could be of higher practical use.

### 3.2. κ-Ga_2_O_3_ Films on n-GaN/Sapphire Templates: Structural Characteristics, Electrical Properties and Deep Traps, Recombination Properties

The HVPE growth of Ga_2_O_3_ on (0001) GaN/sapphire templates at temperatures exceeding 500 °C consistently leads to the formation of orthorhombic κ-Ga_2_O_3_. Its structural properties were studied in some detail in our earlier papers [41,42]. In Ref. [42], we grew κ-Ga_2_O_3_ films of varying thickness at the fixed temperature of 570 °C with growth rates of 3 μm/h, and measured their structural characteristics. The films with a thickness up to 15 μm were grown in a single run. After that thickness was exceeded, the strain build-up in the film commonly led to its detachment from the substrate. However, if the film had a thickness of 15 μm or less, it was cooled down, then heated up to the growth temperature, and additional growth was performed under the same growth conditions as for the thinner films; the overall thickness could be greatly increased.

The reason for the films delaminating is the stress induced by the mismatch of the lattice parameters and the mismatch of the thermal expansion coefficients exceeding a certain value; this is mainly related to the difference in the thermal expansion coefficients and the rate of cooling. If the critical value of stress has not been exceeded, the stress during cooling is partly relieved by the possible generation of dislocations and microcracks, some of which can be revealed by SEM. This partial relief of strain facilitates the growth of thicker films in consecutive growth processes. However, with that, the nucleation of new κ-Ga_2_O_3_ layers on existing κ-Ga_2_O_3_ films can proceed via the formation of multiple nucleation centers; this can result in a texture-like growth with intergrain boundaries and the consequent broadening of FWHM.

For each growth run of θ-2θ XRD scans, the XRD FWHM values of the symmetric 004 and skew-symmetric 206 reflections, the bowing radius of the sample, and the ϕ-scans for the asymmetric reflection values while rotating the sample around the direction normal to the growth plane in order to determine the presence of rotational nanodomains [19] were measured. These measurements were complemented by surface morphology mapping in SEM, MCL spectra measurements, and, for the thickest sample studied, by the EBIC imaging and diffusion length measurements. The thickness of the films was measured using SEM imaging of the cleaved surfaces. Then, θ-2θ XRD scans showed that the films were pure κ-Ga_2_O_3_ with (001) orientation [42]. The FWHM of the symmetric 004 reflection of the relatively thin films with a thickness up to 13 μm, which were prepared in one run, gradually decreased from 24′ to 8.5′; further, the FWHM of the 206 reflection was approximately constant at 12–14′. The thin films were seriously strained, as manifested by the small bowing radius of 4–20 m [42]. For thicker samples grown in several runs, the FWHM of the symmetric reflection was the lowest for the sample with a thickness of 23 μm (9′); the width of the asymmetric reflections did not vary much and further increases in the skew-symmetric reflections and the ϕ-scans around the (001) axis showed characteristic periodic peaks every 60° due to the presence of rotational nanodomains [42].

The FWHM of the XRD reflections is the widely accepted measure of dislocation density of screw and mixed dislocations, which determine the FWHM of symmetric reflections, and of edge dislocations, which determine the FWHM of symmetric reflections [42]. The dislocation densities estimated from the experimental FWHM values according to [56], assuming that FWHM is determined by dislocations, together with the total dislocation density are shown in Table 1. It should be noted that the real dislocation density can be lower than that shown in Table 1, if other defects contribute to the FMHW increase. The range in dislocation densities that is acceptable for proper device operation depends on the device in question and generally is the matter of experimental study.

The surface of the films was mostly flat, but occasionally showed the presence of rather large approximately circular regions where, instead of continuous film, one observed pyramidal microcrystals that were not fully merged [41]. HVPE growth is a process that is characterized by a high growth rate, which requires very careful optimization in order to achieve good morphology. This is the reason why the films studied contained some density of not fully merged regions (their density is around 10^3^ cm^−2^ in the 20-μm films). When increasing the number of growth runs and increasing the thickness of the films, there was a strong increase in the FWHM of both types of X-ray reflections and a strong increase in the bowing radius, signifying a serious strain relief. For the thickest film, which was 86 μm, the ϕ-scans no longer demonstrated a periodic structure, but pointed to the existence of a texture-like structure. The regions with pyramidal microcrystals that were not fully merged were observed in the thick 86 μm films where, however, some growth hexagonal hillocks were observed. Their density was not high and it strongly varied from place to place. For the regions free from such defects, the surface looks quite flat in the secondary electron mode of SEM.

Whether or not the formation of rotational nanodomains was fully suppressed is not totally clear, because the domain structure in the ϕ-scans could be masked by the misorientation of the grains in the texture. Here, detailed transmission electron microscope studies would be necessary. The results of the structural studies are summarized in Table 1. For samples S4 and S6 from Table 1, detailed electrical, photoelectrical, MCL spectra and deep trap measurements, morphology imaging by SEM, and EBIC studies were performed.

**Figure 8:**
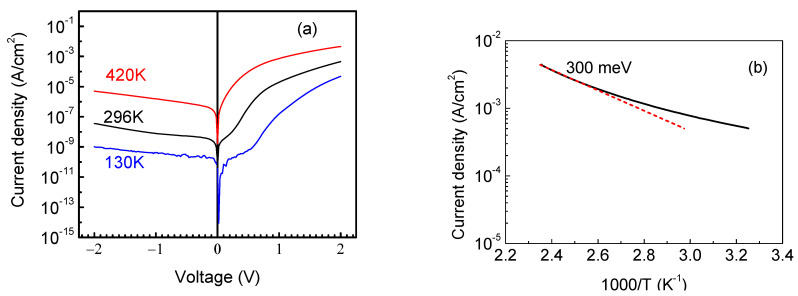
(**a**) I-V characteristics at three different temperatures; (**b**) the temperature dependence of forward current at +2 V.

The current–voltage characteristics for the S4 sample with a thickness of 23 μm are shown for three measurement temperatures in Figure 8a. At room temperature, the ideality factor in the forward direction is essentially higher than unity (2.6), indicating the presence of a high resistivity layer at the surface (the series resistance in forward direction is 2.5 × 10^5^ Ω). The ideality factor decreases to 1.8 and the series resistance decreases to 3 × 10^4^ Ω at 420 K. Figure 7b depicts the temperature dependence of the forward current at +2 V, giving the temperature dependence of the series resistance and the approximate position at which the Fermi level is pinned in the part of the sample limiting the forward current (~0.3 eV).

**Figure 9:**
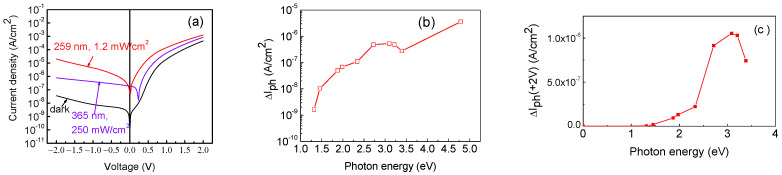
(**a**) I-V characteristic in the dark, with 365 nm LED illumination, and 259 nm LED illumination; (**b**) reverse photocurrent spectrum at −1 V; (**c**) forward photocurrent spectrum at 2 V.

The sample shows a measurable photocurrent in both the forward and reverse directions. Figure 9a presents the dark current, the current with sub-bandgap excitation with 3.4 eV photons, and the photocurrent with above-bandgap excitation with photons of 4.8 eV energy. The above-bandgap photosensitivity is much higher than the sub-bandgap photosensitivity (keep in mind that the optical output power density for the LED with a peak photon energy of 4.8 eV (wavelength 259 nm) was only 1.2 mW/cm^2^, while for the set of LEDs used for sub-bandgap excitation, the optical power density was 250 mW/cm^2^). The spectral dependence of the reverse photocurrent at −1 V (current under illumination minus dark current) is shown in Figure 9b. Optical thresholds near 1.3 eV and 2.3 eV can be clearly seen. Interestingly, the photocurrent is quite pronounced in the forward direction, showing the same optical thresholds (Figure 9c).

**Figure 10:**
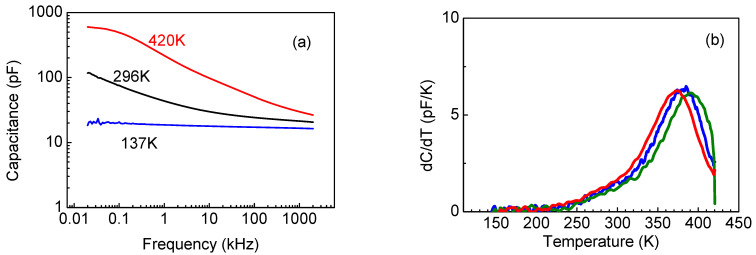
(**a**) C-f characteristics at three temperatures; (**b**) dC/dT for several measurement frequencies: 20 Hz (red), 30 Hz (blue) and 50 Hz (olive).

The capacitance versus frequency C-f dependencies at room temperature, 420 K, and 130 K are shown in Figure 10a. At a high temperature, the C-f characteristic displays a low frequency step that is indicative of the presence of deep traps that can respond to these low probing frequencies. The temperature dependence of the capacitance derivative by temperature, d*C*/d*T*, at these low frequencies shows a distinct peak whose position shifts to lower temperatures as the frequency decreases (Figure 10b presents the data for several frequencies). Standard admittance spectroscopy analysis [46,47] gives the depth of the traps responsible for the peak at 0.7 eV, with an electron capture cross-section *S_n_* of 10^−15^ cm^2^; meanwhile, C-V measurements at these high temperatures and low frequencies yield a trap concentration of 1.2 × 10^17^ cm^−3^ in the near surface submicron region of the film [41]. At high frequencies, the 0.7 eV centers cannot respond to the probing frequency in capacitance measurements; meanwhile the region in which these traps are dominant behaves as a dielectric layer whose capacitance is switched in series with the less resistive part of the film, in which shallower donors with levels near 0.3 eV and a concentration of 6 × 10^15^ cm^−3^ are prevalent [41].

**Figure 11:**
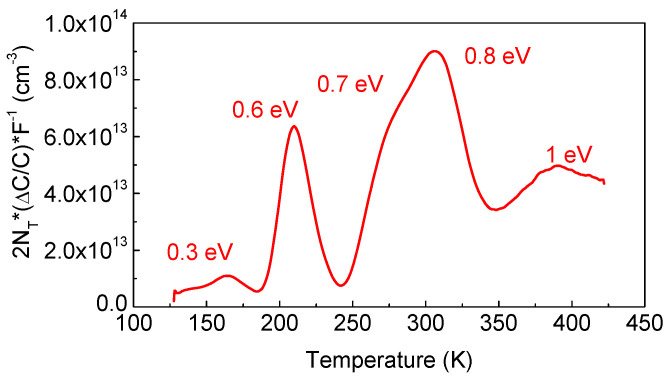
DLTS spectrum with pulsing from −1 V to 2 V, time windows 1.5 s/15 s.

DLTS spectra measured at 100 kHz probe the part of the space charge region where the Fermi level is pinned near *E_c_* − 0.3 eV (depth above ~2 μm [41]) to reveal the presence of electron traps with energy levels at *E_c_* − 0.3 eV, 0.6 eV, 0.7 eV, 0.8 eV, and 1 eV in concentrations in the order of 10^13^–10^14^ cm^−3^ (see Figure 11, in which the *y*-axis is the product of the usual DLTS signal Δ*C* = *C*(*t*1) − *C*(*t*2), normalized by the steady-state capacitance *C*, Δ*C*/*C*, and multiplied by the concentration of centers determining the steady-state width of the space charge region, N_T,_ and by the correlation function of the DLTS spectrometer *F*^−1^, 2*N_T_*Δ*C*/*C* × *F^−^*^1^ [47]) (where *t*1 and *t*2 are the time windows for which the DLTS spectrum was obtained, *N_T_* was taken as the concentration determined from high-frequency C-V measurements). For the temperatures corresponding to the peaks in the spectra, the magnitude of the peaks in such coordinates gives the concentrations of the respective traps without taking into account the so-called λ-correction [47].

**Figure 12:**
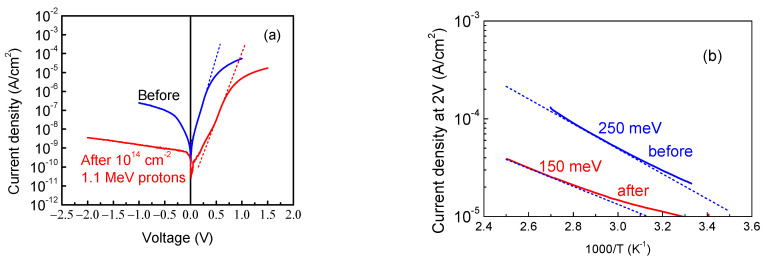
(**a**) room temperature I-Vs before and after proton irradiation; (**b**) the temperature dependence of forward current before and after irradiation.

**Figure 13:**
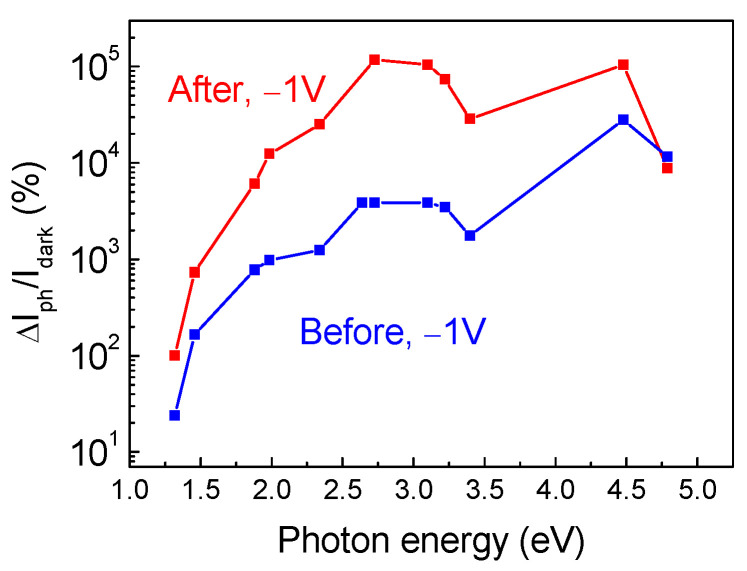
Photocurrent at −1 V normalized by dark current for measurements before and after irradiation.

The behavior of the thickest studied sample yet, S6, was, in fact, very similar to that of the 23 μm thick sample S4. The I-V characteristics showed a somewhat better ideality factor of 1.8, but still a high series resistance (10^6^ Ω at room temperature) (Figure 12a). The temperature dependence of the forward current still showed an activation energy close to that observed in the sample S4 (0.25 eV) (Figure 12b). The photocurrent spectra still showed prominent optical thresholds near 1.3 eV and 2.3 eV (Figure 13 shows the spectral dependence of the current at −1 V under illumination when normalized by the dark current, ΔI_ph_/I_dark_), with the spectral dependence of the forward current possessing the same character.

**Figure 14:**
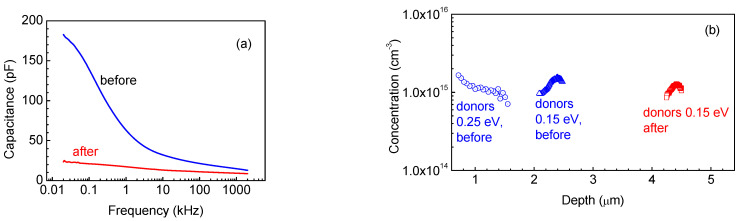
(**a**) C-f characteristics before and after irradiation; (**b**) concentration profiles before and after irradiation.

**Figure 15:**
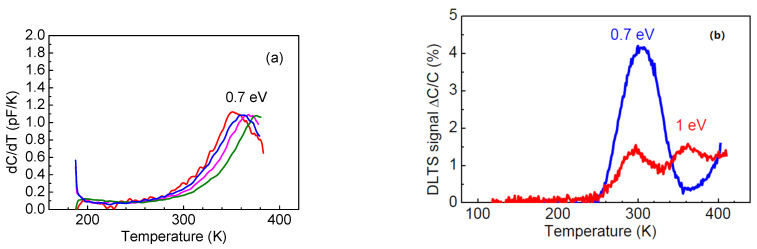
(**a**) Low-frequency dC/dT after irradiation (measurements at frequencies of 20 Hz (red line), 30 Hz (blue line), 50 Hz (magenta line), 100 Hz (olive line)); (**b**) DLTS spectra before and after irradiation.

However, certain differences in the behavior of the thinner sample, S4, could be observed. The C-f characteristic showed a prominent low-frequency step already at room temperature (Figure 14a). Admittance spectra measurements indicated the existence of deeper donors with levels near *E_c_* − 0.25 eV and shallower donors *E_c_* − 0.15 eV, whose concentration profiles calculated from C-V profiling at 90 Hz and 10 kHz are shown in Figure 14b. However, at low frequencies and high temperatures, we still detected high-density of 0.7 eV traps, as demonstrated by Figure 15a. In DLTS spectra of this sample, we observed only one deep trap with level *E_c_* − 0.7 eV (see Figure 15b). The temperature position of the peak in Figure 15b is similar to that of the dominant *E_c_* − 0.8 eV peak in the DLTS spectra of sample S4, suggesting that these could be similar traps.

We also irradiated the 86 μm κ-Ga_2_O_3_ film using the suppressed impact of the rotational nanodomains with 1.1 MeV protons and the fluence of 10^14^ cm^−2^. The main effects of the irradiation are illustrated by Figure 12, Figure 13, Figure 14 and Figure 15. Irradiation led to a strong decrease in the reverse current and increase in the series resistance (up to 3.3 × 10^6^ Ω). It decreased the absolute value of the photocurrent, but, as the decrease in the dark current overpowered the decrease in the photocurrent, the ratio of the photocurrent to dark current (signal-to-noise ratio in photodetectors) was measurably increased (Figure 13). In C-f characteristics, the overall capacitance decreased and the low frequency step corresponding to the deeper 0.25 eV donors completely vanished. This is also seen in the change in the activation energy in the temperature dependence of the forward current (0.15 eV instead of 0.25 eV) (Figure 12b). The concentration profiles calculated from C-V characteristics showed that the density of all the centers responding to the probing frequency of 10 kHz was so decreased that the entire top 4.5 μm of the irradiated sample was depleted of carriers, even at 0 V bias on the Schottky diode (Figure 14b); this indicates that all the shallow centers present in this region before irradiation were removed by subjecting the film to 10^14^ cm^−2^ 1.1 MeV proton irradiation. This thickness is close to the estimated range of 1.1 MeV protons in Ga_2_O_3_. DLTS spectra measurements after irradiation show the emergence of another center observed in the thinner 23 μm sample S4, the *E_c_* − 1 eV trap. However, one should note that these measurements refer to a depth close to 4 μm that was not probed before irradiation.

**Figure 16:**
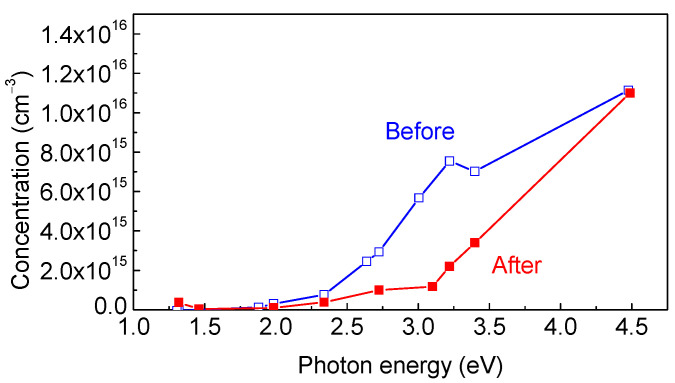
LCV spectra measured before and after irradiation.

Thus, the main effect of proton irradiation on the studied thick κ-Ga_2_O_3_ film with a presumably suppressed formation of rotational nanodomains consists of suppressing the contribution of about 10^15^ shallow donors in the top 4.5 μm of the sample in order to provide free electrons, either as the result of compensation by deep acceptors or by passivation. We tried to determine whether additional deep acceptors were introduced by protons by measuring the concentration profiles in the dark and under illumination using the LCV technique [44,49]. The respective LCV spectra before and after irradiation are compared in Figure 16. It should be noted that for the sample before irradiation, the LCV spectrum is similar to the photocurrent spectrum, with the two major optical ionization thresholds of ~1.3 eV and 2.3 eV, and the main contribution to compensation, being provided by the 2.3 eV centers whose density is in the order of 10^16^ cm^−3^. The 2.3 eV centers have been shown to possess a barrier for the capture of electrons, as follows from the failure to quench their signal after the light was turned off by the application of high forward bias [45,49]. After irradiation, these centers are suppressed, and the major role is overtaken by deep acceptors with an optical ionization threshold of 3.1 eV. Both centers are characterized by the existence of a high barrier for electron capture. The actual mechanism of carrier removal has yet to be understood. Unfortunately, theoretical studies of the electronic states of different defects in κ-Ga_2_O_3_ have not been conducted; this is in contrast to β-Ga_2_O_3_ and α-Ga_2_O_3_ polymorphs (see e.g., [7,8,21,45]), which makes the interpretation of experimental results more difficult. Ii is strange in that, in all three polymorphs, the acceptor defects with optical ionization thresholds of 2 eV to 3 eV seem to play an important role despite the fact that their actual structures are different.

Whatever the exact mechanism involved in the carrier removal of κ-Ga_2_O_3_ is, it can be noted that the measured removal rate is considerably lower than that for β-Ga_2_O_3_ films and crystals with a similar level of n-type doping [9].

**Figure 17:**
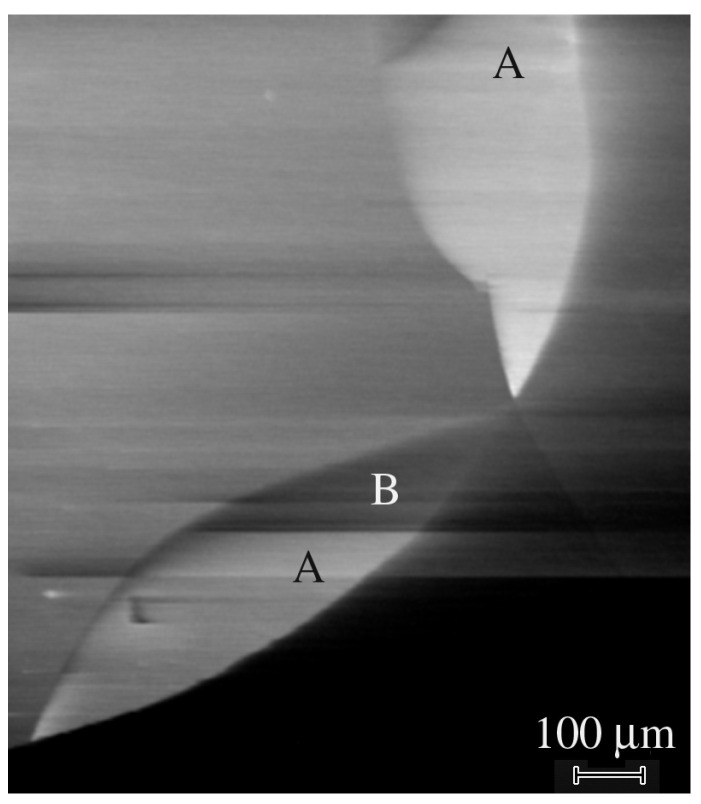
EBIC image of fragment of the Schottky diode formed on the sample S6.

The typical EBIC image of the fragment of the Schottky diode formed on this film is shown in Figure 17. First of all, it should be mentioned that in all images, bright and dark lines can be seen, indicating the presence of small areas with a strong contrast and a slow current decay. In addition, relatively large areas (~a few hundred microns) with varying BIC contrasts can be revealed (Figure 17). Thus, it is seen that in the areas marked by A, the collected current is noticeably larger than that in most of the diode, while in the area B, it is noticeably lower and is practically equal to the current outside the diode. As the measured current, as discussed below, decreases with an increase in serious resistance, it can be assumed that the effective resistance between the Ohmic contact and these regions differs. For example, the region B may be connected to the Ohmic contact via very high resistance. An examination of the secondary electron mode reveals some cracks; however, their number is noticeably lower than the number of boundaries between the regions with varying EBIC contrasts. The surface is rather flat with a small number of regions that consist of microcrystal agglomerations; thus, as the XRD scans point to the existence of a texture-like structure in the thickest film, S6, it is reasonable to assume that this texture-like structure consists of crystallites that create the varying EBIC contrasts. It could be assumed that the crystallite boundaries, some cracks, and the remnant rotational domains, if they still survive in this very thick HVPE grown sample, contribute to the variation in the series resistance. One more factor is the presence of the high-resistivity layer in the top portion of the film, caused by the enhanced concentration of deeper traps.

**Figure 18:**
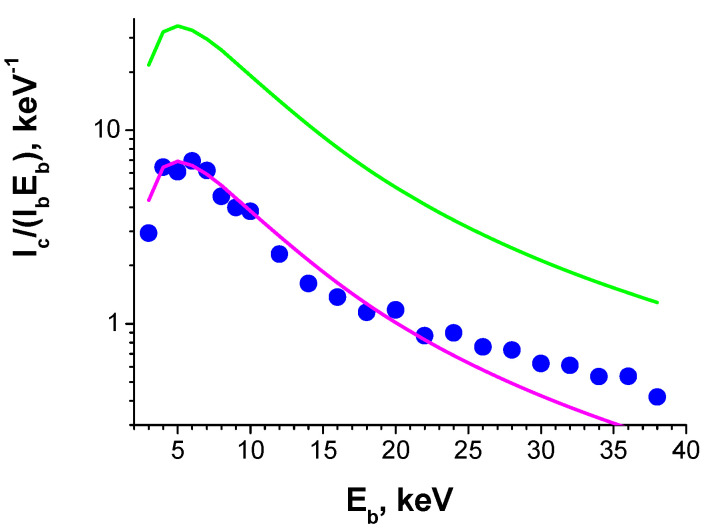
Measured (blue symbols) and calculated (lines) normalized collected current dependencies on beam energy *E_b_*. Magenta line is the simulated dependences and the green one is calculated with η/*E_i_* = 0.05 eV^−1^.

The diffusion length in the S6 sample was estimated by fitting the collected current dependence on beam energy *E_b_* [50,57] with the calculated one. The collected current is calculated using Equation (1). As an example, the measured dependence, together with the fitted one, is presented in Figure 18. The diffusion length is mainly determined by the normalized current decay with beam energy, which allows it to be estimated rather well. The diffusion length in the regions with varying contrasts is practically the same, being in the range of 80 to 100 nm. Proton irradiation also does not practically affect the diffusion length. It should be noted that it is the first measurement of diffusion length in κ-Ga_2_O_3_. In [58], the diffusion length was estimated using the voltage at which the photoresistor gain starts deviating from linearity, assuming that there is some relation between the corresponding electric field and the critical field; below this, the diffusion processes mainly dominate the minority carrier transport. The value of 250–350 nm was obtained, which is not too far away from the values obtained in the present work.

As seen in Figure 17, the collected current differs in different regions. In addition, even in bright regions, the collected current normalized to the beam energy exceeds the beam current by less than 10 times (Figure 18). To estimate the expected collected current value, it should be taken into account that the maximum current value is mainly determined by the η/*E_i_*, where η is the portion of the beam energy absorbed inside the semiconductor and *E_i_* is the mean energy for a formation of an electron–hole pair. For β-Ga_2_O_3_, η/*E_i_* is about 0.05 eV^−1^ [53]. As for κ-Ga_2_O_3_, η should slightly decrease due to the higher density and *E_i_* should decrease due to the smaller bandgap of κ-Ga_2_O_3_, compared with β-Ga_2_O_3_. Thus, it can be assumed that for κ-Ga_2_O_3_, the η/*E_i_* value is close to that for β-Ga_2_O_3_. The magenta line in Figure 18 shows the result of fitting the experimental dependence with the diffusion length of 80 nm. The fitting is reasonably good, but there are some problems. First, the actual normalized current is about five times lower than the current calculated with expected the η/*E_i_* = 0.05 eV^−1^ (the result of the latter calculation is shown in the green line). To explain this decrease, it should be taken into account that the measured current can be expressed as follows:

(3) $$I=I_{0}\{exp[\frac{e}{nkT}(V-R_{s}I)]-1\}-I_{c},$$

where *I*_0_ is the saturation current, *R_s_* is the series resistance, *n* is the ideality factor depending on the contribution of the recombination current, *k* is the Boltzmann constant, *T* is the temperature and *I_c_* is the collected current in the structure with negligible *R_s_*. As follows from (3), large *R_s_* should decrease the measured induced current. As shown above, the presence of the compensated layer in the top portion of the film strongly increases the series resistance and could result in the observed discrepancy. This high series resistance is the result of the presence of the ~2 μm thick top layer with a high density of deep traps (AS spectroscopy suggests the density of ~10^17^ cm^−3^ *E_c_* − 0.7 eV centers), thus leading to strong compensation. The different EBIC contrasts can be explained by the different resistance of crystallites within the film. The situation can be aggravated by the additional effect of crystallite boundaries and possibly the remnant contribution of the nanodomains. In addition, the width of the SCR that needs to be assumed for the good quality of fitting corresponds to a charge density >10^17^ cm^−3^, which is much higher than the free carrier concentration obtained by C-V profiling; however, it is close to the trap concentration in the layer that is about 2 μm from the surface [42] (see above). The effective width of the SCR could be different in C-V and EBIC measurements, since some conducting channels could exist. If the thickness of these channels is very small, they may affect the current, but practically not affect the capacitance. These channels can be formed by carrier tunneling, via traps along the domain boundaries or other extended defects. Such channels could also explain the slower decay in the current at large *E_b_* values in comparison with the calculated ones, as seen in Figure 18. Such a slowdown in the decay was not observed in β-Ga_2_O_3_ [51].

**Figure 19:**
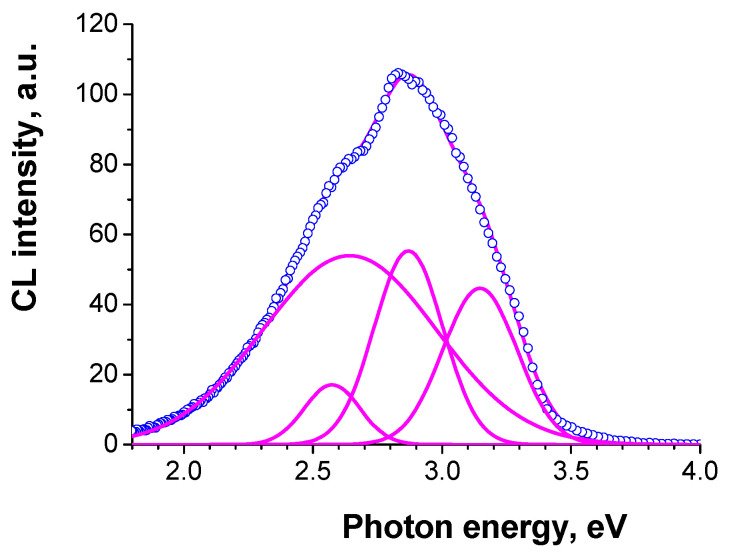
Typical CL spectrum measured on the flat surface of sample S6 with a beam energy of 10 keV (blue symbols). The emission bands and the resultant simulated spectrum are shown with magenta lines.

**Figure 20:**
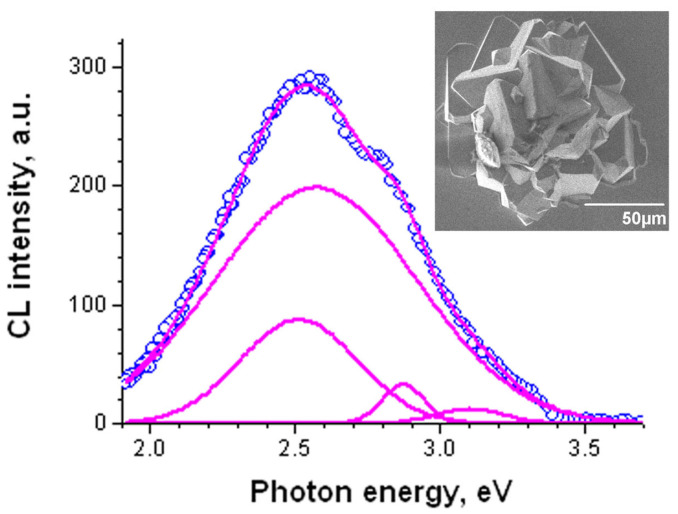
CL spectrum measured on the crystallite agglomerate (shown in the inset) with a beam energy of 10 keV (blue symbols). The emission bands and the resultant simulated spectrum are shown with magenta lines.

The CL spectra measured on the flat regions consist of emission bands with energies of 2.57, 2.64, 2.87 and 3.15 eV (Figure 19). The spectra measured on the S4 and S6 samples are very similar, with only the relationship between the component intensities being slightly different. However, on some conglomerates of crystallites, the spectrum is different (Figure 20). It consists of 2.51, 2.57, 2.87 and 3.1 bands, thus, only the 2.64 eV band disappears and a new 2.51 eV band appears. It can be also assumed that in the conglomerates of crystallites, the bands with the smaller energy (2.57 and 2.64) are shifted to the lower energies due to changes in the strain or quantum confinement effect. It should be also pointed out that the spectrum maximum of conglomerates shifts to lower energies.

The proton irradiation did not practically affect the CL spectra. Thus, it can be concluded that the concentration of the main radiative recombination centers is not practically affected by irradiation.

## 4. Summary and Conclusions

As mentioned in the Introduction, the main interest in the κ-Ga_2_O_3_ polymorph comes from the presence of a strong spontaneous polarization that far exceeds the electrical polarization in III-Nitrides. This offers the possibility of creating two-dimensional carrier gases at suitable interfaces and also of using the ferroelectric properties of κ-Ga_2_O_3_. The presence of rotational nanodomains in κ-Ga_2_O_3_ does not preclude the observation of the beneficial effects related to high polarization. The studies that have demonstrated the advantageous polarization properties and ferroelectric properties of ε/κ-Ga_2_O_3_ have been performed for films with a poor structural quality that definitely contain rotational nanodomains as dominant structural defects. However, the useful performance of structures with two-dimensional carriers at the interface is strongly affected by the mobility of 2D carriers and depends on minimizing the detrimental impact of the structural defects that cause the local breakdown of 2D conductivity. As the structural perfection of κ-Ga_2_O_3_ films was, until recently, quite poor, the theoretical predictions of the performance of different heterostructures with κ-Ga_2_O_3_ were mostly hypothetical. The reports of actual observations of 2D carriers’ effects in the heterojunctions involving κ-Ga_2_O_3_ are scarce. In fact, the experimental demonstration of the formation of 2DHG at the κ-Ga_2_O_3_/AlN interface reported above is one of the few examples of its kind. Such a heterojunction does not seem promising for use in devices because of the poor crystalline quality of the film and the poor conductivity of both the AlN and the lightly doped κ-Ga_2_O_3_ films, which make it difficult to realize Ohmic contacts. Other issues to be clarified include stabilizing the proper polarity of the κ-Ga_2_O_3_ and AlN, using free-standing n-type AlN to facilitate the proper vertical current flow and using free-standing n-GaN instead of AlN, since the difference in the spontaneous polarization of κ-Ga_2_O_3_ and GaN is much higher than that for AlN.

More straightforward applications should be based on heterojunctions of κ-(Al_x_Ga_1−x_)_2_O_3_ with κ-Ga_2_O_3_, for which the starting point is the preparation of good-quality κ-Ga_2_O_3_. We have shown that such films can be grown using HVPE on GaN/sapphire templates. Judging by the FWHM of the reflections in HRXRD, the best crystalline quality is achieved for epilayers with a thickness of 23 μm, grown in two long consecutive growth runs. Such films still display the characteristic 60° periodicity in the ϕ-scans for asymmetric HRXRD reflection, indicative of the formation of rotational domains that are not fully suppressed. We believe that this is due to the presence of regions in which the film has not completely merged and consists of separate columns of κ-Ga_2_O_3_ [41]. The growth of even thicker films leads to the relief of strain via the formation of a sort of texture that has misoriented grains and grain boundaries that consist of dislocations, as indicated by the increase in the FWHM of HXRD reflections.

This process becomes predominant for the thickest 86 μm film. It is not clear whether the formation of rotational domains is fully suppressed in this structure, but as their formation is the result of the film trying to accommodate the strain [19], one expects that, as in the ELOG growth [19], the nanodomains should become much less of a problem. Further studies are necessary to prove this unambiguously. Electrical measurements show that the in-plane resistance of both the 23 μm and the 86 μm films is still high because of the presence of strong compensation in the upper ~2 μm of the layers; this is due to a high density of 0.7–0.8 eV traps and of deep acceptors, with optical ionization thresholds of 1.3 eV and 2.3 eV. The similarity of behavior for the 23 μm and the 86 μm films suggests that such near surface defects form during the late stages of growth or during the cooling of the film. The traps are most likely responsible for the poor in-plane conductance of thin κ-Ga_2_O_3_ films and for the short lifetimes of nonequilibrium charge carriers, as determined by EBIC experiments in which no EBIC signal could be obtained for the continuous parts of the 23 μm film [41]. The optimization of the HVPE growth suppressing the formation of these defects should bring the in-plane conductivity of the films and the lifetime of charge carriers to much higher levels.

The diffusion length of excess carriers in κ-Ga_2_O_3_ films was measured for the first time. The diffusion length was 80–100 nm, i.e., much shorter than in typical β-Ga_2_O_3_ films [9]. The effects of the irradiation of 1.1 MeV protons on the electrical properties, deep trap spectra, and recombination properties of these films were studied for the first time. The observed carrier removal rate of about 10 cm ^−1^ is considerably lower than that for similarly doped β-Ga_2_O_3_ films, which could be an advantage for κ-Ga_2_O_3_-based devices when used in harsh radiation environments.

## Figures and Tables

**Table 1:** Structural characteristics of κ-Ga_2_O_3_ films grown by HVPE on (GaN (0001)/sapphire.

Sample No	Number of Growth Runs	Thickness (μm)	FWHM (arcMin)			ρ_s_ Screw, cm^−2^	ρ_e_ Edge, cm^−2^	ρ Total, cm^−2^
			004	206	206 (twist)	004	206 (twist)	
S1	1	10	24′	18′	36′	4 × 10^9^	4 × 10^10^	4.4 × 10^10^
S2	1	13	9′	12′	28′	1.7 × 10^9^	3 × 10^10^	4.7 × 10^10^
S3	2	20	12′	12′	24′	2 × 10^9^	2.6 × 10^10^	2.8 × 10^10^
S4	2	23	16′	14′	26′	2.1 × 10^9^	2.8 × 10^10^	3 × 10^10^
S5	4	60	20′	34′	48’	3.3 × 10^9^	7 × 10^10^	7.3 × 10^10^
S6	6	86	60′	60′	90′	1 × 10^10^	13 × 10^10^	14 × 10^10^

## Data Availability

Data are contained within the article.
